# CN54gp140: product characteristics, peclinical and clinical use - recombinant glycoprotein for HIV immunization

**DOI:** 10.1186/1742-4690-9-S2-P351

**Published:** 2012-09-13

**Authors:** D Katinger, S Jeffs, F Altmann, A Cope, P McKay, N Almond, E Sandström, B Hejdeman, G Biberfeld, C Nilsson, D Hallengärd, B Wahren, T Lehner, M Singh, DJ Lewis, C Lacey, R Shattock

**Affiliations:** 1 Polymun Scientific GmbH, Klosterneuburg, Austria; 2 Imperial College, London, UK; 3 Universität für Bodenkultur, Vienna, Austria; 4 HPA-NIBSC, Potters Bar, UK; 5 Karolinska Institutet, Stockholm, Sweden; 6 Swedish Institute for Communicable Disease Control, Solna, Sweden; 7 King’s College London at Guy's Hospital, London, UK; 8 Lionex Diagnostics & Therapeutics GmbH, Braunschweig, Germany; 9 University of Surry, Guildford, UK; 10 University of York, York, UK

## Background

The usefulness of HIV envelope proteins for vaccine design is widely accepted since the RV144 HIV-1 prime-boost vaccine trial. It is assumed that a trimeric structure close to the natural form of the HIV envelope is preferable.

## Methods

We have expressed the soluble form of the HIV envelope of the C/B' strain 97/CN/54 in CHO cells. The production process consisted of a large-scale fed-batch fermentation, an antibody-based affinity chromatography plus additional purifying steps. CN54gp140 was extensively characterized for purity and identity. All glycosylation sites were characterized by mass spectrometry. Immunogenicity and safety were evaluated in mice, rabbits, mini-pigs, sheep as well as non-human primates. The antigen was formulated for clinical phase I studies in Tris buffer (MUCOVAC I, HIVIS07) in HEC gel (MUCOVAC I) as well as chemically conjugated to hsp70 (MUVAPRED).

## Results

The vaccine antigen candidate CN54gp140 proved to be of high purity and long-term stability. The immune response was strongest with i.m. application whereas the mucosal routes (i.vag., i.n.) were less immunogenic. Safety was demonstrated in animal models as well as in the clinical phase I studies MUCOVAC I, MUVAPRED and HIVIS07.

## Conclusion

CN54gp140 is a highly immunogenic trimeric envelope protein which can be manufactured in sufficient quality and quantity for clinical application. It proved to be immunogenic in several animal models. Finally, it is well tolerated in several formulations and combinations in humans.

